# Ultralow Lattice Thermal Conductivity of the Random Multilayer Structure with Lattice Imperfections

**DOI:** 10.1038/s41598-017-08359-2

**Published:** 2017-08-15

**Authors:** Pranay Chakraborty, Lei Cao, Yan Wang

**Affiliations:** Department of Mechanical Engineering, University of Nevada, Reno, Nevada 89557 USA

## Abstract

Randomizing the layer thickness of superlattices (SL) can lead to localization of coherent phonons and thereby reduces the lattice thermal conductivity *κ*_*l*_. In this work, we propose strategies that can suppress incoherent phonon transport in the above random multilayer (RML) structure to further reduce *κ*_*l*_. Molecular dynamics simulations are conducted to investigate phonon heat conduction in SLs and RMLs with lattice imperfections. We found that interfacial species mixing enhances thermal transport across single interfaces and few-period SLs through the phonon “bridge” mechanism, while it substantially reduces the *κ*_*l*_ of many-period SLs by breaking the phonon coherence. This is a clear manifestation of the transition from incoherent-phonon-dominated to coherent-phonon-dominated heat conduction in SLs when the number of interface increases. In contrast, interfacial species mixing always increases the *κ*_*l*_ of RMLs owing to the dominance of incoherent phonons. Moreover, we found that doping a binary RML with impurities can reduce *κ*_*l*_ significantly, especially when the impurity atom has an atomic mass lower or higher than both of the two base elements. This work reveals the critical effect of lattice imperfections on thermal transport in SLs and RMLs, and provides a unique strategy to hierachically suppress coherent and incoherent phonon transport concurrently.

## Introduction

The quest for waste heat recovery technologies has raised continued interest in thermoelectric (TE) materials, which can convert waste heat into reusable electricity directly. TE devices can also work in the reversed mode, in which electricity can be used to move heat and thus achieve solid-state cooling. This is advantageous over conventional air-conditioners with moving pumps and condensers. The figure of merit ZT of TE materials is

1 $$ZT=\frac{\sigma S{T}^{2}}{{\kappa }_{e}+{\kappa }_{l}},$$

in which *σ* is the electrical conductivity, *S* is the Seebeck coefficient, T is the absolute temperature, *κ*_*e*_ is the electronic thermal conductivity, and *κ*_*l*_ is the lattice thermal conductivity. Since *σ* and *κ*_*e*_ are usually strongly coupled via the Wiedemann-Franz’s law, tuning *κ*_*l*_ has been a widely adopted scheme to increase the ZT of TE materials^1, 2^.

Various nanostructures have been theoretically proposed or experimentally demonstrated to have a substantially reduced *κ*_*l*_ compared to their bulk counterparts, for example, nanowires^3, 4^, nanocomposites^5, 6^, superlattices (SL)^7, 8^, nanomeshes^9, 10^, and pillared thin films^11–13^. Typically, the low *κ*_*l*_ of the above structures partially or completely stems from the extensive surface and interface scatterings in the nanostructures, which can be understood by the Casimir picture^14^, i.e., classical size effect. For SLs, nanomeshes, and pillared thin films, their phonon dispersion relations can be significantly different from their bulk counterparts, which also contribute to the low *κ*_*l*_ found in these structures. Semiconductor alloys, for example, Si/Ge and Bi_2_Te_3_/Sb_2_Te_3_^15–17^, have also received substantial attention owing to their low *κ*_*l*_ and relatively lower cost for manufacturing. Furthermore, considerable efforts have been devoted to combining different phonon scattering mechanisms to hierarchically scatter phonons of different wavelength to achieve a *κ*_*l*_ below the random-alloy limit. For example, Kim *et al*. embedded ErAs nanoparticles in In_0.53_Ga_0.47_As alloys, so that short-wavelength phonons are scattered by point defects and mid-to-long-wavelength phonons are scattered by the embedded nanoparticles^18^. Biswas *et al*. extended this strategy even further by creating an all-scale hierarchical architecture, in which atomic-scale point defects, nanoscale inclusions, and mesoscale grain boundaries scatter short-, mid-, and long-wavelength phonons, respectively^19^. This has led to one of the highest ZTs for TE materials so far. In addition, Hu *et al*. found ultralow *κ*_*l*_ of Si/Ge superlattice nanowires, which arises from the combined effects of interface scattering and lateral surface scattering^20^.

Recently, phonon localization has been found to suppress coherent phonon transport in multilayers with random layer thickness^21–24^, of which the structure will be referred to as random multilayer (RML) in this manuscript. It has been reported that perfect RMLs with short average layer thickness can have remarkably lower *κ*_*l*_ than binary alloys^22^ and superlattices with rough interfaces of the same composition^22, 24^, suggesting their promising application as TE materials. Coherent phonons are often the major heat carriers in structurally perfect SLs at low to moderate temperatures^21^. In RMLs, however, *κ*_*l*_ is substantially reduced owing to the localization of coherent phonons^21^. Mu *et al*. have demonstrated substantial reduction in the *κ*_*l*_ contributed by coherent phonons through hierarchical scattering or localization of coherent phonons with amorphous or rough surfaces, multiple interfaces, and randomly positioned Si layers^25^. On the other hand, as demonstrated in ref. 21, heat conduction in RMLs is mainly contributed by incoherent phonons, which have relatively short mean-free-path and wavelength. This inspires us to investigate whether we can further reduce the *κ*_*l*_ of RMLs by nanostructuring to minimize incoherent phonon transport, for example, by introducing interfacial species mixing or lattice impurities, which have been demonstrated to reduce the *κ*_*l*_ of materials effectively. In particular, incoherent phonons, which typically have short wavelength, dominate thermal transport in RMLs. Since the atomic mass difference induced by alloying or impurity doping preferably scatters short-wavelength phonons^26^, it can possibly be utilized to further reduce the *κ*_*l*_ of RMLs. This motivates us to study doped RMLs in this work, which can provide us with new strategies for hierarchically manipulating both coherent and incoherent phonon transport in materials. Moreover, there are almost always various degrees of structural imperfections in multilayered structures fabricated in lab using any deposition technique^27, 28^. It is thereby of practical importance to fully understand their influence on phonon transport, transmission, and localization.

In addition to the issues discussed above, understanding phonon transport across single interfaces and multiple parallel interfaces is essential for understanding the overall heat transfer behavior in RMLs and SLs. However, contradictory results exist in literature regarding the effect of interface disorder on phonon transport across single interfaces or multiple interfaces. For instance, interface roughness has been found to reduce the thermal boundary resistance (TBR) of single interfaces^29–31^ and that of sandwiched amorphous layers^32^, while many other groups have reported remarkably increased TBR or reduced *κ*_*l*_ of SLs by interface disorder^22, 24, 33, 34^. The reduction in the TBR of single interfaces has been attributed to the phonon “bridge” effect, in which the mixed interfacial region serves as a “bridge” for the phonon spectra of the two sides of the interface. On the other hand, the reduction in the *κ*_*l*_ of SLs is attributed to the breaking of phonon coherence by the interface disorder. It can be expected that coherent phonon transport should play a major role in such discrepancy, while a direct evidence has not been reported yet. In this work, we strive to understand the effect of structural disorders, namely, random layer thickness, interfacial species mixing, and impurities on phonon transport in SLs and RMLs. We conduct molecular dynamics simulations to systematically investigate the above issues and provide strategies on further reducing the *κ*_*l*_ of RMLs.

## Methodology

**Figure 1:**
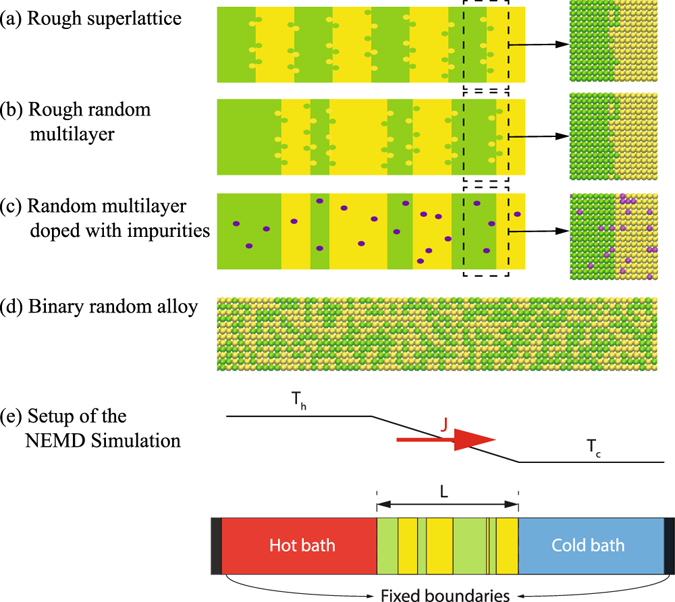
Schematic of the structures simulated in this work and the corresponding atomic structure: (**a**) rough superlattice; (**b**) rough random multilayer; (**c**) random multilayer doped with impurities; (**d**) binary random alloy. (**e**) shows the setup of the NEMD simulation.

We conduct non-equilibrium molecular dynamics (NEMD) simulations to investigate phonon heat conduction in the structures shown in Fig. 1, namely, SL with rough interfaces (rough SL), rough RML, RML doped with impurities (doped RML), and binary random alloy. Specifically, interface roughness is modeled as interfacial species mixing. The structures are mostly composed of conceptual atoms with an atomic mass of *m* = 40.0 g/mol or *m* = 90.0 g/mol, which we will refer to as m40 and m90 atoms. For doped RML, as shown in Fig. 1c, impurity atoms are randomly doped to the RML lattice to replace m40 or m90 atoms. The impurity has an atomic mass of *m* = 22.5 g/mol, *m* = 60.0 g/mol, or *m* = 160 g/mol, which will be referred to as m22.5, m60, and m160 atoms, respectively. The RML structures are created by replicating the conventional fcc unit cell (UC) with a lattice constant of 5.278 *Å* in all three dimensions. The m40 and m90 layers are stacked along the [100] lattice direction to form the SLs or RMLs. We adopt the algorithm that is described in ref. 21 to generate the thickness of the layers in RMLs. The layer thickness follows a truncated Gaussian distribution under the constraint that the average layer thickness is the same as the corresponding SL. This guarantees a fair comparison between the phonon transport in SLs and RMLs. The interatomic interactions are described by the two-body Lennard-Jones potential,

2 $${\varphi }_{ij}({r}_{ij})=4\varepsilon [{(\frac{\sigma }{{r}_{ij}})}^{12}-{(\frac{\sigma }{{r}_{ij}})}^{6}],$$

in which *ϕ*_*ij*_ is the pairwise interaction potential energy, *r*_*ij*_ is the distance between atom *i* and atom *j*, *ε* is the potential well depth, and *σ* is the zero-potential-energy pair separation. We set the value of the parameters for the Lennard-Jones potential to be *ε* = 0.1664 eV and *σ* = 0.34 nm. The cutoff radius for truncating interatomic interactions in the molecular dynamics simulation is chosen as *r*_*c*_ = 2.5*σ*. Our rationale for using the Lennard-Jones potential rather than a more realistic one, e.g., the Tersoff potential, is that it can capture the key physics of coherent phonon effects in multilayered structures^21^ with substantially lower computational cost.

Figure 1e shows the setup of the NEMD simulation domain. The structure of interest is sandwiched between two 327-*Å*-long heat baths. It has been found in ref. 21 that heat baths of this length is necessary to obtain converged results of *κ* for the Lennard-Jones system studied here. The heat baths are maintained at a temperature of *T*_*h*_ = 33 K (hot bath) and *T*_*c*_ = 27 K (cold bath), respectively. The simulation time step is chosen as Δ*t* = 1 fs. We use the LAMMPS Molecular Dynamics Simulator^35^ to conduct NEMD simulations. At the beginning of the simulation, the periodic boundary condition is applied to all three dimensions and each atom is assigned a random velocity following the Gaussian distribution. The average kinetic energy of the atoms corresponds to a temperature of 5 K. Subsequently, the entire simulation domain is relaxed in the NPT ensemble at zero pressure and a temperature of 30 K for 200 ps. After the NPT structural relaxation process, we apply the fixed boundary condition to the heat flow direction by freezing an 11-*Å*-thick layer of atoms at both ends of the simulation domain. A length of 11 *Å* guarantees that there is no cross-boundary energy transfer between the two ends of the simulation domain. Finally, atoms in the hot bath are rescaled to a temperature of 33 K and those in the cold bath are rescaled to 27 K in every simulation step for a total time period of 40 ns, during which we collect the heat current and temperature distribution of the structure. The lattice thermal conductivity *κ*_*l*_ of the structure is calculated as *κ*_*l*_ = *JL*/[*A*_*c*_(*T*_*h*_ − *T*_*c*_)], in which *A*_*c*_ and *L* are the cross-sectional area and the length of the structure, respectively. The heat transfer rate *J* is calculated as $J=(\frac{d{E}_{h}}{dt}+\frac{d{E}_{c}}{dt})\mathrm{/2}$, in which *E*_*h*_ and *E*_*c*_ are the kinetic energy that has been added to or subtracted from the hot bath or cold bath by the time *t*. At steady state, *E*_*h*_ and *E*_*c*_ should be a linear function of *t*. Therefore, we fit the steady-state *E*_*h*_-*t* and *E*_*c*_-*t* curves using the linear least squares regression method to obtain *J*, and the standard deviation in *J* is used to estimate the error bars for *κ*_*l*_.

The vibrational density of states (vDOS) are calculated as the Fourier transform of the atomic velocity-autocorrelation functions averaged over the group of atoms of interest. Specifically, the normalized vDOS is calculated as

3 $$g(\omega )={\int }_{-\infty }^{\infty }dt\frac{{\sum }_{i=1}^{N}\langle {{\bf{v}}}_{i}(t)|{{\bf{v}}}_{i}\mathrm{(0)}\rangle }{{\sum }_{i=1}^{N}\langle {{\bf{v}}}_{i}\mathrm{(0)|}{{\bf{v}}}_{i}\mathrm{(0)}\rangle }{e}^{i\omega t},$$

where *g* is the vDOS, *ω* is the angular frequency of phonons, **v**_*i*_ is the velocity vector of atom *i*, 〈**v**_*i*_(*t*)|**v**_*i*_(0)〉 denotes the velocity-autocorrelation function, and *N* denotes the number of atoms in the group. *ω* is related to the phonon frequency *f* via *ω* = 2*πf*. In our work, molecular dynamics simulations are conducted for 20,000 steps in the NVE ensemble to obtain the autocorrelation function.

### Availability of materials and data

The datasets generated during and/or analysed during the current study are available from the corresponding author on reasonable request.

## Results and Discussion

### Effect of interface roughness

We first investigate the effect of interface roughness in the form of interfacial species mixing on thermal transport in SLs and RMLs. Interfacial species mixing is an inevitable source of lattice defect in multilayered structures fabricated by current layer deposition techniques. Its effect on phonon heat conduction has been investigated extensively and, in general, it was found to reduce the *κ*_*l*_ of SLs substantially. The reduction in *κ*_*l*_ has been attributed to the breaking of phonon coherence by the interface disorder^22, 34^. Herein we focus on a general comparison between the *κ*_*l*_ of perfect SLs, rough SLs, perfect RMLs, and rough RMLs.

**Figure 2:**
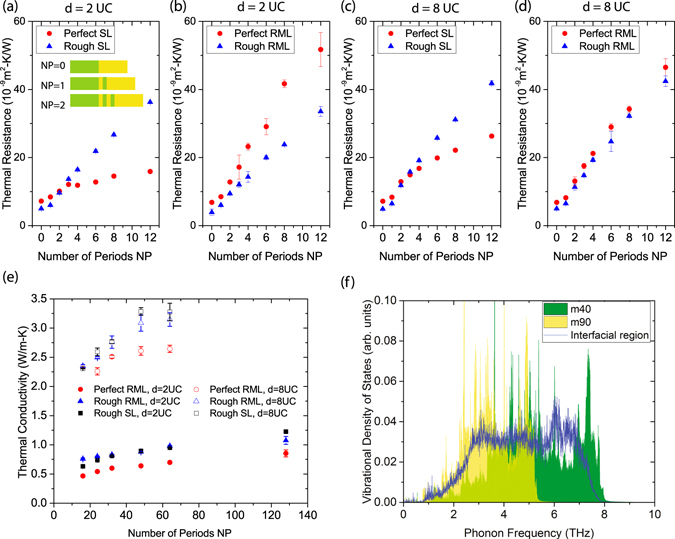
(**a**–**d**) Thermal resistance of perfect SL, rough SL, perfect RML, or rough RML as a function of the number of periods NP for an average layer thickness of *d* = 2 UC (panel a and b) or *d* = 8 UC (panel c and d). The inset of (**a**) illustrates the meaning of NP. In particular, NP = 0 corresponds to a single interface between m40 and m90. (**e**) The lattice thermal conductivity *κ*_*l*_ as a function of NP. (**f**) The vibrational density of states of the m40, m90, and interfacial regions of a rough RML.

First, we compute the thermal resistance *R* of SLs and RMLs with smooth or rough interfaces as a function of the number of periods (NP). Specifically, *R* is calculated as *R* = *A*_*c*_(*T*_*h*_ − *T*_*c*_)/*J*. In addition to SLs and RMLs, we also study the case of NP = 0, which corresponds to a direct contact between a heat bath composed of m40 atoms and one composed of m90 atoms, i.e., a single interface between m40 and m90. Schematics of the structures with NP = 0, 1, and 2 can be found in the inset of Fig. 2a.

In Fig. 2a and b, we show the *R* of SLs and RMLs with an average layer thickness *d* = 2 UC. As for the effect of interface species mixing on thermal transport in SLs, the most striking feature we can observe is the crossover between *R*_perfect-SL_ and *R*_rough-SL_ occurring at NP = 2. Specifically, interface species mixing reduces the thermal resistance when the SL has no more than 2 periods, while it increases *R* when the SL has more periods. This elucidates the contradictory observations regarding the effect of interface disorder on phonon transport across single interfaces and SLs^22, 24, 29–31, 33, 34^. We attribute the crossover to the transition from incoherent-phonon-dominated heat conduction regime to coherent-phonon-dominated one when NP increases. Coherent phonons are formed due to the constructive interference between the incident and reflected phonon waves at multiple, parallel interfaces. For single interfaces and SLs with few periods, incoherent phonon dominates heat transfer because no or few phonon interferences can happen due to the limited number of interfaces. In these structures, incoherent phonon transport across each individual interface can be enhanced by interface species mixing through the phonon “bridge” mechanism^36^. As shown in Fig. 2f, the phonon spectra of the m40 and m90 region, which are denoted by the green and yellow colors, respectively, mainly overlap in a narrow frequency range of 3–5 THz. However, the interfacial region in RMLs or SLs with interfacial species mixing has a vDOS (blue curve) that largely overlaps with both the vDOS of the m40 and that of the m90 regions, which serves as a phonon “bridge” facilitating phonon transmission. In contrast, for SLs with more periods, phonon interference can happen more frequently and coherent phonons start to dominate heat conduction. In this case, interface disorder breaks phonon coherence, thus reducing the chance for constructive phonon interference. As a result, the *R* of the SL is increased.

Different from SLs, coherent phonons are localized in RMLs owing to the random layer thickness^21^. Accordingly, incoherent phonons dominate heat conduction in RMLs. Figure 2b clearly demonstrates the characteristics of incoherent phonon transport behavior in RMLs, in which the *R* of rough RMLs is always lower than that of the corresponding perfect RMLs. Similar to the cases of single interfaces and few-period SLs, the reduced *R* is also caused by the phonon “bridge” effect discussed above.

For SLs with *d* = 8 UC, Fig. 2c shows a clear crossover of the *R* of perfect SLs and rough ones at NP = 2. For RMLs with *d* = 8 UC, however, the difference between perfect and rough ones is rather trivial, as displayed in Fig. 2d. This is merely a result of the dominance of the bulk thermal resistance of each individual layer over the TBR between layers. Indeed, the thermal resistance of RMLs, despite of the interface condition, increases almost linearly with the total length (proportional to NP) of the RML. This suggests that a simple thermal circuit model can be used for RMLs, i.e.,

4 $$R={\rm{NP}}\times (\frac{d}{{\kappa }_{m40}}+\frac{d}{{\kappa }_{m90}})+\mathrm{(2}{\rm{NP}}+\mathrm{1)}\times {\rm{TBR}},$$

of which the first term on the right-hand side corresponds to the bulk thermal resistance of individual layers and the second term arises from the TBR between layers. Obviously, the bulk resistance term dominates *R* for RMLs with relatively thick layers (*d* = 8 UC); thus interface species mixing cannot have a significant impact on the overall *R* even though it reduces TBR. Moreover, as discussed in ref. 21, anharmonic phonon-phonon scatterings inside each individual layer can kill the phonon coherence in long-period SLs. The resulting dominance of incoherent phonons in a long-period SL will also cause a dominance of the bulk resistance term in the overall thermal resistance *R* of the SL. Therefore, it can be expected that interface species mixing does not affect the overall *R* of long-period SLs significantly.

We also compare the *κ*_*l*_ of SLs and RMLs with NP up to 128 for *d* = 2 UC cases and up to 64 for *d* = 8 UC cases. As we can see in Fig. 2e, the *κ*_*l*_ of rough SLs is higher than that of rough RMLs when the structures contain many layers (NP = 128 for *d* = 2 UC and NP ≥ 24 for *d* = 8 UC). The reason is that even though interface species mixing breaks phonon coherence significantly, there can still be a certain amount of coherent phonons surviving in rough SLs, while they are localized in rough RMLs. Therefore, rough SLs can have a higher *κ*_*l*_ than rough RMLs. However, the *κ*_*l*_ of rough RMLs with *d* = 2 UC is higher than that of the corresponding rough SLs for NP = 16 and NP = 24. This is because there are many layers containing only 1-UC-thick m40 or m90 layers, while interface species mixing effectively convert the whole 1-UC-thick layer into alloy. As a result, several such layers can sometimes connect as one continuous alloy layer rather than individual m40 or m90 layers, which reduces the number of interfaces in the RML. Consequently, the *κ*_*l*_ of such rough RMLs might be higher than that of the corresponding SL, which always have distinct interfaces between layers.

Based on the above analysis, we can conclude that for RMLs with short average layer thickness, it is beneficial to make the interfaces as smooth as possible, which helps to minimize the *κ*_*l*_. The above results also suggest that strategies that can hinder the transport of short-wavelength incoherent phonons can further reduce the *κ*_*l*_ of RMLs. This leads us to investigate the effect of impurities (point defects) on the *κ*_*l*_ of RMLs, which is well-known to scatter short-wavelength phonons effectively.

### Effect of impurity

In this section, we investigate the thermal transport in RMLs doped with impurities in the form of point defects. Specifically, we simulate RMLs composed of m40 layers and m90 layers, both doped with 10% foreign atoms randomly. The dopant has an atomic mass of 22.5 g/mol, 60.0 g/mol, and 160.0 g/mol, which will be referred to as m22.5, m60, and m160, respectively. These atoms interact with m40 and m90 atoms based on the same Lennard-Jones potential described in the Methodology section of this manuscript. We also simulate binary random alloy, which is composed of randomly mixed 50% m40 and 50% m90 atoms.

**Figure 3:**
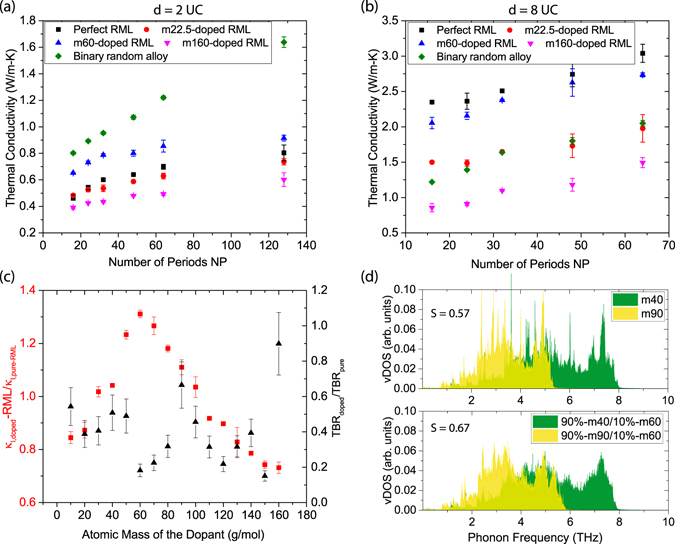
(**a**,**b**) The lattice thermal conductivity *κ*_*l*_ of perfect RMLs, RMLs doped with impurities with an atomic mass of 22.5 g/mol, 60.0 g/mol, or 160.0 g/mol, and binary random alloy of 50% m40/50% m90. The RMLs in (**a**) have an average layer thickness *d* = 2 UC while those in (**b**) have *d* = 8 UC. (**c**) Left axis: the relative *κ*_*l*_ of doped RMLs (*d* = 2 UC) with respect to that of pure RMLs. Right axis: the TBR (normalized by the TBR of the pure m40-m90 interface) between m40 and m90 segments doped with 10% impurities with various atomic mass. (**d**) The vibrational density of states of m40 and m90 elements and those of m40 and m90 crystals doped with 10% m60 atoms.

As shown in Fig. 3a and b, the *κ*_*l*_ of binary random alloys is generally higher than that of perfect RMLs with *d* = 2 UC, while it is lower than that of perfect RMLs with *d* = 8 UC. The problem with binary random alloy is that even though short-wavelength phonons are scattered strongly, long-wavelength phonons can travel a long distance in the alloy and thus carry a considerable amount of heat^37^. In contrast, short-period RMLs can scatter short-wavelength incoherent phonons strongly by the interfaces and localize long-wavelength coherent phonons effectively by the random layer thickness, which can lead to a lower *κ*_*l*_ than binary random alloys with the same composition. We can also see that adding m22.5 and m160 reduces the *κ*_*l*_ of RMLs in all cases simulated in this work. In particular, m160-doping can reduce the *κ*_*l*_ of RMLs by up to 33% and 53% for RMLs with *d* = 2 UC and *d* = 8 UC, respectively. It is reasonable because the short-wavelength incoherent phonons are scattered by these impurities owing to the mass-difference scattering mechanism and the bulk thermal resistance of each layer increases. Nonetheless, adding m60 atoms causes an increase in *κ*_*l*_ for short-period RMLs (*d* = 2 UC), which is unwanted for thermoelectric applications.

To comprehensively understand the effect of the weight of dopants on the *κ*_*l*_ of RMLs, we simulate a series of RMLs doped with impurities of an atomic mass varying from *m*_*im*_ = 10 g/mol to *m*_*im*_ = 160 g/mol. Specifically, 10% of the atoms in the pure RML lattice are randomly selected and their atomic mass is changed to *m*_*im*_. In this way, we can isolate the effect of the atomic mass of the dopants from the effect of the location of doping sites. As we can see in Fig. 3c, of which the red squares are the relative *κ*_*l*_ of doped RMLs with respect to that of pure ones, *κ*_*l*_ increases with *m*_*im*_ first and decreases thereafter. The maximum *κ*_*l*_ occurs at *m*_*im*_ = 60 g/mol, which is the geometric average of the atomic mass of the two base materials, i.e., 40 g/mol and 90 g/mol. Moreover, the *κ*_*l*_ of the doped RML is obviously higher than that of the undoped RML if 40 g/mol < *m*_*im*_ < 90 g/mol. We find that the increase in *κ*_*l*_ of RMLs is caused by the reduction in the TBR of interfaces. The dark triangles in Fig. 3c denote the TBR (normalized by the TBR of the pure m40–m90 interface) of the interfaces between m40 and m90, of which 10% of the atoms are replaced with impurities. As we can see, doping reduces the TBR of the interfaces in the RMLs. In particular, doping impurities with an atomic mass of 60 g/mol can reduce the TBR by as much as 80%. According to Eq. 4, in short-period RMLs, the substantial reduction in TBR can lead to an increase in the overall *κ*_*l*_ of the RML. In contrast, in RMLs with large *d*, the bulk thermal resistance of layers dominates the overall heat transfer. Since the dopants scatter incoherent phonons, which increases the bulk thermal resistance of layers, the overall *κ*_*l*_ is reduced by doping.

To further understand the above observations, we analyze the vDOS of m40 layers and m90 layers in a perfect RML and the vDOS of a m40–m90 RML doped with 10% m60 atoms. The upper panel of Fig. 3d shows the vDOS of pure m40 and m90 regions of the RML. As we can see, the phonon spectrum of m40 is at a higher frequency region than m90, which is due to the lower mass of m40 atoms. The lower panel of Fig. 3d shows the vDOS of the m40 and m90 regions doped with 10% m60 atoms. It can be seen that the overlap between the two phonon spectra is larger than that of the pure m40-m90 pair. To quantify the overlap between the phonon vDOS of the m40 and m90 layers, we calculate the overlap factor *S* using the approach defined in previous work^22, 38^. In particular, *S* varies between 0 for completely non-overlapping phonon spectra and 1 for perfect overlap. Agreeing with the change in TBR, we find that *S* is significantly increased from 0.57 for undoped RML to 0.67 by m60-doping. The increase in *S* is expected to facilitate phonon transport across the interface owing to the phonon “bridge” mechanism discussed above.

It is worth mentioning that even though the *κ*_*l*_ of m60-doped RMLs is higher than that of undoped RMLs for *d* = 2 UC, m60-doping can still reduce incoherent phonon transport within each individual m40 and m90 layer, similar to m22.5 and m160. However, since m60-doping causes a more significant reduction in TBR than the increase in phonon scattering inside the layers, the overall *κ*_*l*_ of the RML still increases. To avoid this, we recommend doping with impurities lighter than the lightest or heavier than the heaviest element of the original RML. In this way, no significant phonon “bridge” effect will occur. Moreover, the *α* = 10% impurity concentration already makes the TE material very heavily doped. In most cases, *α* is lower than 10%, which, as we can expect, will lead to less scattering of incoherent phonons. As a result, the *κ*_*l*_ of the RML will increase.

## Summary

In summary, we conducted nonequilibrium molecular dynamics simulations to investigate phonon transport in random multilayer structures containing lattice imperfections. We found that interface species mixing can increase the *κ*_*l*_ of RMLs, because the mixed interfacial region has an intermediate phonon vDOS between the adjacent layers and thus facilitates phonon transport across the interface through the phonon “bridge” effect. We also observed that the *κ*_*l*_ of rough SL is usually higher than that of the corresponding rough RML. This is because interface disorder does not kill all the phonon coherence and the remaining coherent phonons lead to a higher *κ*_*l*_ of rough SLs than that of rough RMLs. Moreover, we investigated the dependence of total thermal resistance *R* on the number of periods in SLs with smooth and rough interfaces. We revealed that interface roughness reduces the thermal resistance of a single interface or SLs with few periods, while it increases the thermal resistance of SLs containing many periods. The crossover in thermal resistance manifests a transition from incoherent-phonon-dominated heat transfer regime to a coherent-phonon-dominated one. When the SL has few periods, including the case for a single interface, heat conduction is dominated by incoherent phonons, of which the transmission can be enhanced by interface species mixing through the phonon “bridge” effect. In contrast, considerable amount of coherent phonons are formed owing to the interference of reflected and incident phonons at multiple, parallel interfaces in SLs. Interface disorder breaks phonon coherence and thus reduces the *κ*_*l*_ of SLs significantly. Finally, we studied the effect of impurities on thermal transport in RMLs and found that point defects can substantially reduce *κ*_*l*_. However, if the atomic mass of the impurities is between that of the two base materials, *κ*_*l*_ may be increased for RMLs with a short average layer thickness, which is ascribed to a reduction in TBR. This work revealed a novel strategy to hierarchically suppress both coherent and incoherent phonon transport in multilayered structures, which can aid the design of nanostructures with ultralow lattice thermal conductivity.
